# Peach allergen Pru p 1 content is generally low in fruit but with large variation in different varieties

**DOI:** 10.1002/clt2.12034

**Published:** 2021-05-14

**Authors:** Jing Jin, Kexin Gan, Lan Zhao, Huijuan Jia, Yifan Zhu, Xiongwei Li, Zhaowei Yang, Zhengwen Ye, Ke Cao, Zhiqiang Wang, Mingliang Yu, Yuyan Zhang, Zhisheng Ma, Hangkong Liu, Pere Arús, Jaap H. Akkerdaas, Zhongshan Gao, Ronald van Ree

**Affiliations:** ^1^ Allergy Research Center Zhejiang University Hangzhou China; ^2^ College of Agriculture and Biotechnology Zhejiang University Hangzhou China; ^3^ Forest & Fruit Tree Institute Shanghai Academy of Agricultural Sciences Shanghai China; ^4^ State Key Laboratory of Respiratory Disease The First Affiliated Hospital of Guangzhou Medical University Guangzhou China; ^5^ Zhengzhou Fruit Research Institute China Academy of Agricultural Sciences Zhengzhou China; ^6^ Fruit Tree Institute Jiangsu Academy of Agricultural Sciences Nanjing China; ^7^ Shijiazhuang Pomology Institute Hebei Academy of Agriculture and Forestry Sciences Shijiazhuang Hebei China; ^8^ College of Horticulture Northwest A&F University Yangling Shaanxi China; ^9^ IRTA Centre de Recerca en Agrigenòmica CSIC‐IRTA‐UAB‐UB Campus UAB – Edifici CRAG Barcelona Spain; ^10^ Departments of Experimental Immunology and Otorhinolaryngology Amsterdam UMC University of Amsterdam Amsterdam The Netherlands

**Keywords:** ELISA, hypoallergenic varieties, peach allergy, protein quantification, Pru p 1

## Abstract

**Background:**

Pru p 1 is a major allergen in peach and nectarine, and the different content in varieties may affect the degree of allergic reactions. This study aimed to quantify Pru p 1 levels in representative peach varieties and select hypoallergenic Pru p 1 varieties.

**Methods:**

To obtain monoclonal and polyclonal antibodies, mice and rabbits, respectively, were immunized with recombinant Pru p 1.01 and Pru p 1.02. The Pru p 1 levels in fruits from 83 representative peach varieties was quantified by sandwich enzyme‐linked immunosorbent assay (sELISA). nPru p 1 was obtained through specific monoclonal antibody affinity purification and confirmed by Western blot and mass spectrometry. The variable Pru p 1 content of selected varieties was evaluated by Western blot and the expression level of encoding *Pru p 1* genes by quantitative polymerase chain reaction.

**Results:**

A sELISA method with monoclonal and polyclonal antibodies was built for quantifying Pru p 1 levels in peach. Pru p 1 was mainly concentrated in the peel (0.20–73.44 μg/g, fresh weight), being very low in the pulp (0.05–9.62 μg/g) and not detected in wild peach. For the 78 peach and nectarine varieties, Pru p 1 content varied widely from 0.12 to 6.45 μg/g in whole fruit. We verified that natural Pru p 1 is composed of 1.01 and 1.02 isoallergens, and the *Pru p 1* expression level and Pru p 1 band intensity in the immunoblots were in agreement with protein quantity determined by ELISA for some tested varieties. In some cases, the reduced levels of Pru p 1 did not coincide with low Pru p 3 in the same variety in whole fruit, while some ancient wild peach and nectarines contained low levels of both allergens, and late‐ripening yellow flesh varieties were usually highly allergenic.

**Conclusion:**

Pru p 1 content is generally low in peach compared to Pru p 3. Several hypoallergenic Pru p 1 and Pru p 3 varieties, “Zi Xue Tao,” “Wu Yue Xian,” and “May Fire,” were identified, which could be useful in trials for peach allergy patients.

## 1 BACKGROUND

Peach (*Prunus persica* (L.) Batsch) is rich in nutrients beneficial to health. It is a native fruit crop of China but is grown worldwide with high levels of consumption.^1^ However, it is one of the fruits most frequently reported as allergenic, with sensitization prevalence in Europe increasing from 5.4% to 7.9% in 2014, and it poses a potential major risk to some individuals.^2^, ^3^

To date, allergens identified in peach fruit and pollen include Pru p 1 (pathogenesis‐related 10 proteins [PR‐10]), Pru p 2 (TLP), Pru p 3 (nonspecific lipid transfer protein), Pru p 4 (profilin), Pru p 7 (gibberellin‐regulated protein),^2^ Pru p 9 (PR‐1, pollen allergen),^4^ and ENEA (the first four N‐terminal residues, similar to latex Hev b 5), a recently identified allergen.^5^ Of these, the two main allergens Pru p 1 and Pru p 3 together account for more than 95% of peach allergies in Europe and China.^3^, ^6^, ^7^ They induce two different allergy patterns: in central Europe and north China the symptoms are mild and local, such as oral allergy syndrome (OAS) related to Pru p 1, while in southern Europe and China the symptoms are mostly OAS and/or systemic due to Pru p 3.^3^, ^8^ Hypersensitivity caused by Pru p 1 is mostly induced by cross‐reactions with Fagales pollen group 1 allergenic proteins, such as Bet v 1.^9^, ^10^, ^11^

Three naturally occurring isoforms of Pru p 1, Pru p 1.0101, Pru p 1.0201, and Pru p 1.0301, have been identified in the “Earlygold” peach cultivar and their immunoglobulin E (IgE)‐binding efficiency has been studied. Pru p 1.0301 is mainly expressed in peach pollen, and Pru p 1.0101 (DQ251187) and Pru p 1.0201 (KM350692) isoallergens are present in the fruit, with Pru p 1.0201 having the highest binding capacity to IgE compared with Pru p 1.0101 and Pru p 1.0301.^12^, ^13^ This means that Pru p 1.0101 and Pru p 1.0201 can essentially represent the total Pru p 1 content in peach fruit.

Allergen levels in various fruits have been extensively studied to help patients avoid high‐risk varieties, especially in Rosaceae fruits such as apple and peach. The PR‐10 protein levels are highly dependent on the varieties and also influenced by storage conditions and duration.^14^, ^15^, ^16^ LTP and PR‐10 proteins have been most studied due to their high sensitization rate and cross‐reactivity. LTP is stable, mainly concentrated in the peel and the levels vary greatly in different varieties, related to ripening, aroma, and sugar content.^17^, ^18^, ^19^ The content of PR‐10 proteins has been found to be lower than that of LTP in peach, and is usually affected by genotype, storage conditions and storage time.^14^, ^20^ A recent study selected Pru p 3 hypoallergenic varieties after measuring over 100 varieties using sensitive monoclonal antibody ELISA.^19^ Quantification of the Pru p 1 levels is necessary to analyze whether there is a relationship between fruit quality (aroma, sugar content, ripening date) and Pru p 1 content, to evaluate the potential risk level of Pru p 1 and to screen out hypoallergenic varieties for both Pru p 1 and Pru p 3.

The aim of this study was to establish a sandwich enzyme‐linked immunosorbent assay (sELISA) method, to quantify Pru p 1 content in 83 peach varieties and define varieties with low levels of Pru p 1 allergen to benefit peach allergenic patients.

## 2 MATERIALS AND METHODS

### 2.1 Plant materials

Based on peach genetic diversity previously identified in China, a core collection of 19 nectarine and 64 peach varieties (60 cultivated and 4 wild), a total of 83, were selected (Table S1).^21^, ^22^ Most of these varieties were cultivated in an experimental orchard in Jiaxing, Zhejiang Province. Fruits from different varieties were collected in two consecutive years, 2018 and 2019. Total soluble solids (°Brix) content was measured with a digital refractometer (PR‐101α; ATAGO), and peach aroma intensity was classified subjectively as light, medium, or strong (Table S1) based on the Descriptors and Data Standard for Peach.^23^ Peach fruit were pitted and separated into peel, pulp and whole (including peel and pulp). The samples were stored at −40°C.

### 2.2 Preparation of protein extracts and protein determination

Protein extraction was carried out as described previously.^18^ Briefly, 1 g of homogenized material was ground to powder in liquid nitrogen and taken up in Coca’s solution (0.1 M Na_2_CO_3_, 0.1 M NaHCO_3_, 0.1 M NaCl, 2 mM EDTA–Na_2_, 20 mM sodium diethyldithiocarbamate trihydrate), at 1:5 (wt/vol) for pulp and whole samples and 1:10 (wt/vol) for the peel samples. Each peach variety contained three replicate samples. The supernatant was stored at −30°C and used for Pru p 1 quantification within 2 days. Total protein content was determined with the Bradford Assay Kit (Sangon Biotech) according to the manufacturer’s instructions.

### 2.3 Mouse monoclonal antibodies

To produce anti‐Pru p 1.0101 and anti‐Pru p 1.0201 mAbs, four Bal b/c mice were immunized subcutaneously intraperitoneally with 10–40 μg (interval of 10 μg) rPru p 1.0101 and rPru p 1.0201, obtained from previous research.^13^ The mice were boosted five times twice at 2‐week intervals with 50 μg antigens in Incomplete Freund’s adjuvant. Antibody producing hybridoma cells secreting anti‐rPru p 1 monoclonal antibodies were selected by ELISA and Western blot with two isoallergen antigens and peach peel extracts. Antibodies were purified using HiTrap Protein‐A affinity chromatography.

### 2.4 Rabbit polyclonal antibodies

Two New Zealand rabbits were immunized with four subcutaneous boosters with rPru p 1.0101 and rPru p 1.0201. The primary injection was 0.5 mg antigen emulsified in Complete Freund's Adjuvant, and this followed by three booster injections with different intervals 7–14 days and 0.75 mg antigens emulsified in Incomplete Freund's Adjuvant. Antibodies (Rb IgG) specific to the two isoallergens were derived from hyperimmune sera and purified by protein A column. Two pAbs P1 anti‐rPru p 1.0101 and P2 anti‐rPru p 1.0201 were obtained and detected by direct ELISA using Pru p 1 and Pru p 3 as antigens to test their capacity and specificity. These antibodies were produced by HuaAn Biotech Ltd. (authorization for use of animals no. SCXK 2016‐0004 and SCXK 2017‐0004).

### 2.5 Affinity chromatography and liquid chromatography–mass spectrometry

Protein (5 mg) extracted from peach peel (cv. “Jin Shuo”) was loaded onto 20 ml gravity columns (Sangon Biotech) previously coupled with 6 mg of A2‐D8 antibody. MAb‐bound Pru p 1 isoforms were eluted with a glycine buffer (0.1 M, pH = 2.4) and then dialyzed to phosphate‐buffered saline (PBS) (pH = 8.3). Peach peel extract and eluate samples were analyzed by sodium dodecyl sulfate–polyacrylamide gel electrophoresis (SDS‐PAGE) and Western blot. Liquid chromatography–mass spectrometry (LC‐MS/MS) after in‐gel trypsin digestion was used for identity‐matching of the purified protein to deduced allergens from peach extract.

### 2.6 sELISA quantification of Pru p 1 allergen

Monoclonal and polyclonal antibodies specific for the two Pru p 1 isoallergens were used to find the suitable pair for sELISA. A0‐A7‐G11 was selected as the capturing antibody due to its high affinity to both isoallergens, with bound proteins detected by biotinylated pAbs P1 and P2 (1:1 mixed) in conjunction with horseradish peroxidase‐conjugated streptavidin (Amsersham Bioscience).

Recombinant Pru p 1 was obtained in our laboratory through cloning, heterologous expression, and purification using the AKTA explorer (GE Healthcare) system, on a HisTrap FF crude column (GE Healthcare). Detailed methods on Pru p 1 have been included in a previous article.^13^ Pru p 1 (rPru p 1.0101 and rPru p 1.0201, 1:1 mixed) was used as the standard protein to construct the response curve for sELISA. Detection was with goat anti‐rabbit immunoglobulin G (IgG) (Sangon Biotech) labeled with peroxidase (1:2000 diluted). To guarantee the precision of the measurement, standard curves were repeated with a twofold serial dilution from 0.12 to 500 ng/ml, with duplication in every batch of four 96‐well plates. The peach samples were diluted with PBS at suitable ratios (peel 1:100, pulp 1:5, whole fruit 1:20) so that the optical density values were within the working range. The calculated Pru p 1 (including Pru p 1.0101 and Pru p 1.0201) content was expressed as microgram per fresh weight (FW) of whole fruit or specific tissue. Detailed ELISA (sELISA) has been published in a previous article.^18^

### 2.7 SDS‐PAGE and immunoblot

Proteins were separated by 12% acrylamide gels and electroblotted (100 V, 350 mA, 1 h) onto polyvinylidene fluoride membrane (0.2 μm; Bio‐Rad) in a mini Trans‐Blot electrophoretic transfer cell (Bio‐Rad). After blocking with 5% (wt/vol) skimmed milk for 1 h, the membrane was washed three times in TBST, then incubated with 5 μg/ml polyclonal P1 and P2 (used in ELISA as detecting antibodies, 1:1 mixed) for 1 h. After washing, the membrane was incubated for a further hour with goat anti‐rabbit IgG coupled with HRP (1:1500 dilution; Sangon Biotech). Finally, the membrane was incubated for 2 min in the dark with 2 ml chromogenic reagent (Bio‐Rad, Clarity^TM^ Western ECL Substrate) and the chemiluminescence was recorded using the ChemiDoc Imaging system (Bio‐Rad).

### 2.8 Quantitative polymerase chain reaction of *Pru p 1.0101* and *Pru p 1.0201* genes in 10 selected varieties

Total RNA was isolated from whole peach with three independent replications using the Rapid Universal Plant RNA Extraction Kit supplied by Hua Yue Yang Biotechnology, following the manufacturer’s instructions. Three replicates of the amplification and a negative control were done for all samples. The concentration of total RNA was determined by the absorbance at 260 nm (A260) using a NanoDrop spectrometer. First‐strand complementary DNA (cDNA) was synthesized using HiScript® II Q Select RT SuperMix for quantitative polymerase chain reaction (qPCR) (+gDNA wiper) (Vazyme, China). Real‐time PCR reactions were in a total volume of 20 μl, containing 0.4 μl (10 μmol/L) of each primer, 1 μl diluted cDNA, and 10 μl 2 × ChamQ Universal SYBR qPCR Master Mix (Vazyme). The primers selected were *Pru p 1.0101*, *Pru p 1.0201*, and *ACTIN*, based on our previous study.^24^ The qPCR conditions were: 30 s at 95°C, followed by 45–50 cycles of 10 s at 95°C for template denaturation, 20 s at 60°C for annealing and 20 s at 72°C for extension and fluorescence measurement. The specificity of amplification was confirmed by melting curve analyses and the correct size of the amplification products confirmed by the presence of a single band of expected size for each primer pair in electrophoresis gels.

### 2.9 Statistical analyses

Quantitative data were expressed as mean ± SE by Prism 6.0 (GraphPad Software). Significant differences among groups were assessed using the Kruska–Wallis nonparametric test. All statistical tests with *p* < 0.05 were considered as significant. Real‐time PCR data were presented according to the comparative method (2^−ΔΔ*C*t^), where Δ*C*_t_ is the difference in threshold cycles for the target (*C*_t_ sample) and reference (*C*_t_ *ACTIN*). Excel and Prism 6.0 were used for qPCR statistical analyses and figure plotting.

## 3 RESULTS

### 3.1 Selection of mAbs and pAbs

**FIGURE 1:**
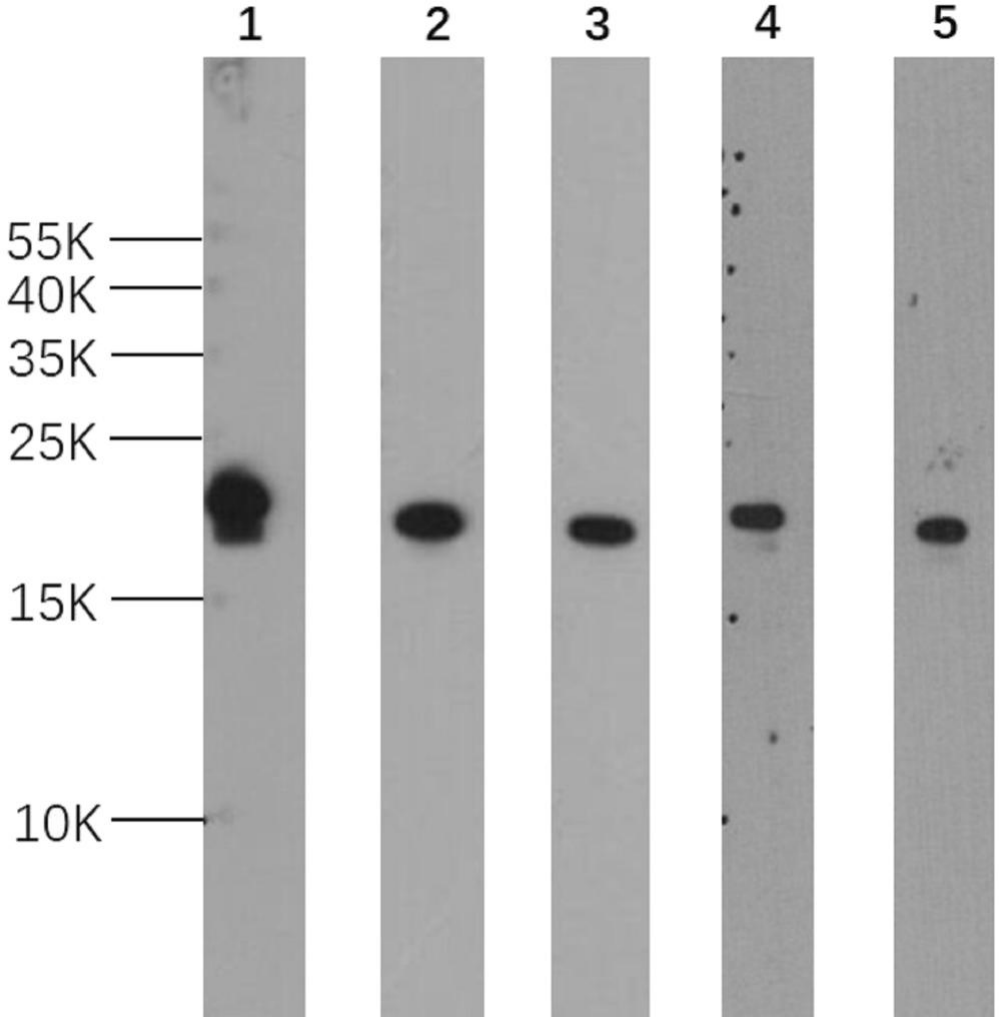
Western blot of selected mAbs specific to rPru p 1. Five anti‐Pru p 1 mAbs. mAbs A0‐A7‐G11 (lane 1), B6‐A1‐B11 (lane 2), and A2‐D8 (lane 3) were produced against rPru p 1.0101; C7‐C4 (lane 4), 5‐D10 (lane 5) were produced against Pru p 1.0201. Five micrograms peach crude extract were added in each lane

Screening by ELISA against rPru p 1.01 and rPru p 1.02 resulted in five mAbs and two pAbs. The mAbs A0‐A7‐G11, B6‐A1‐B11, A2‐D8, and pAb P1 were obtained against Pru p 1.0101, and two mAbs, C7‐C4, 5‐D10, and the pAb P2 against Pru p 1.0201. All mAbs detected Pru p 1 at 17 kDa in Western blot (Figure 1). Preliminary testing indicated that mAb A0‐A7‐G11 had high binding capacity and specificity, making it a suitable capturing antibody. As binding of the polyclonal antibodies P1 and P2 was similar for both isoallergens, they were mixed at a ratio 1:1 as the detecting antibodies. Pair matching and antibody specificity tests were also carried out (Tables 1 and 2).

**TABLE 1:** Monoclonal antibody pair matching test for sandwich ELISA (OD at A450 nm)

mAb	Bio‐P1	Bio‐P2
**A0‐A7‐G11**	**1.66**	**1.51**
B6‐A1‐B11	1.17	1.64
C7‐C4	0.72	1.10
A2‐D8	0.04	0.26
5‐D10	0.19	0.35

*Note:* Coated with 100 μl of 3 μg/ml mAb, rPru p 1 1 μg/ml (rPru p 1.0101 and rPru p 1.0201, 1:1 mixed), detector pAb 100 μl of 3 μg/ml.

Abbreviations: ELISA, enzyme‐linked immunosorbent assay; OD, optical density.

**TABLE 2:** Specificity of antibodies for Pru p 1 (OD at A450 nm)

Antigen	rPrup 1.0101	rPrup 1.0201	rPru p 1.0101:rPru p 1.0201 (1:1 mix)	Peach peel extract	nPru p 3	rMal d 1
Antibody						
A0‐A7‐G11	1.37	1.53	1.89	1.28	0.04	0.12
P1	1.91	1.24	2.03	1.24	0.05	0.70
P2	1.52	1.53	1.79	1.27	0.11	1.33
P1:P2 (1:1 mix)	1.74	1.86	1.91	1.31	0.23	1.22

*Note:* Coated with 100 μl of 3 μg/ml A0‐A7‐G11, antigen 100 μl at 1 μg/ml (peach peel extract 100 μl), detector pAb 100 μl at 3 μg/ml (P1 and P2 1:1 mixed).

Abbreviation: OD, optical density.

### 3.2 Dose–response standard curve

**FIGURE 2:**
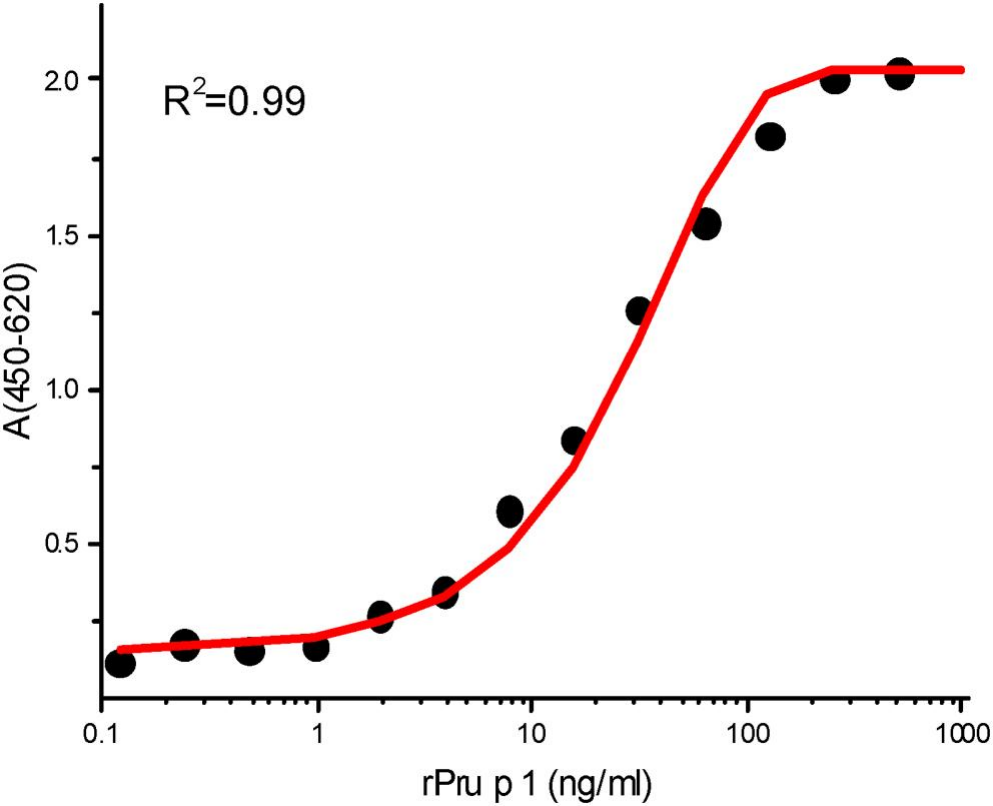
Standard curve of rPru p 1 by sandwich enzyme‐linked immunosorbent assay (sELISA). Dose–response standard curve of rPru p 1 in sELISA with mAb A0‐A7‐G11 as the capture antibody and biotinylated PAbs P1 and P2 as the detector antibody

The dose–response standard curve, obtained with rPru p 1.0101 and rPru p 1.0201 (1:1 mixed), ranged from 0 to 500 ng/ml with a linear section between 4 and 32 ng/ml (Figure 2). The intra‐assay coefficient of variation ranged from 2.88% to 7.25% and that of interassay from 1.96% to 7.58%, indicating high sensitivity, accuracy and reproducibility (Table 3).

**TABLE 3:** The percent recovery and the coefficients of variation of sandwich ELISA

Spiked rPru p 1 (ng/ml)	Intra‐assay (*n* = 6)			Inter‐assay (*n* = 3)			
	Measured (ng/ml)	SD (ng/ml)	CV (%)	Recovery rate	Measured (ng/ml)	SD (ng/ml)	CV (%)
4	4.54	0.024	6.74	113.5	4.1	0.03	7.58
8	8.09	0.021	4.02	101.1	7.71	0.02	4.96
16	16.05	0.024	2.88	100.3	15.81	0.02	1.96
32	32.53	0.091	7.25	101.6	29.80	0.04	3.04

Abbreviation: ELISA, enzyme‐linked immunosorbent assay.

### 3.3 Natural Pru p 1 purification and protein identity

**FIGURE 3:**
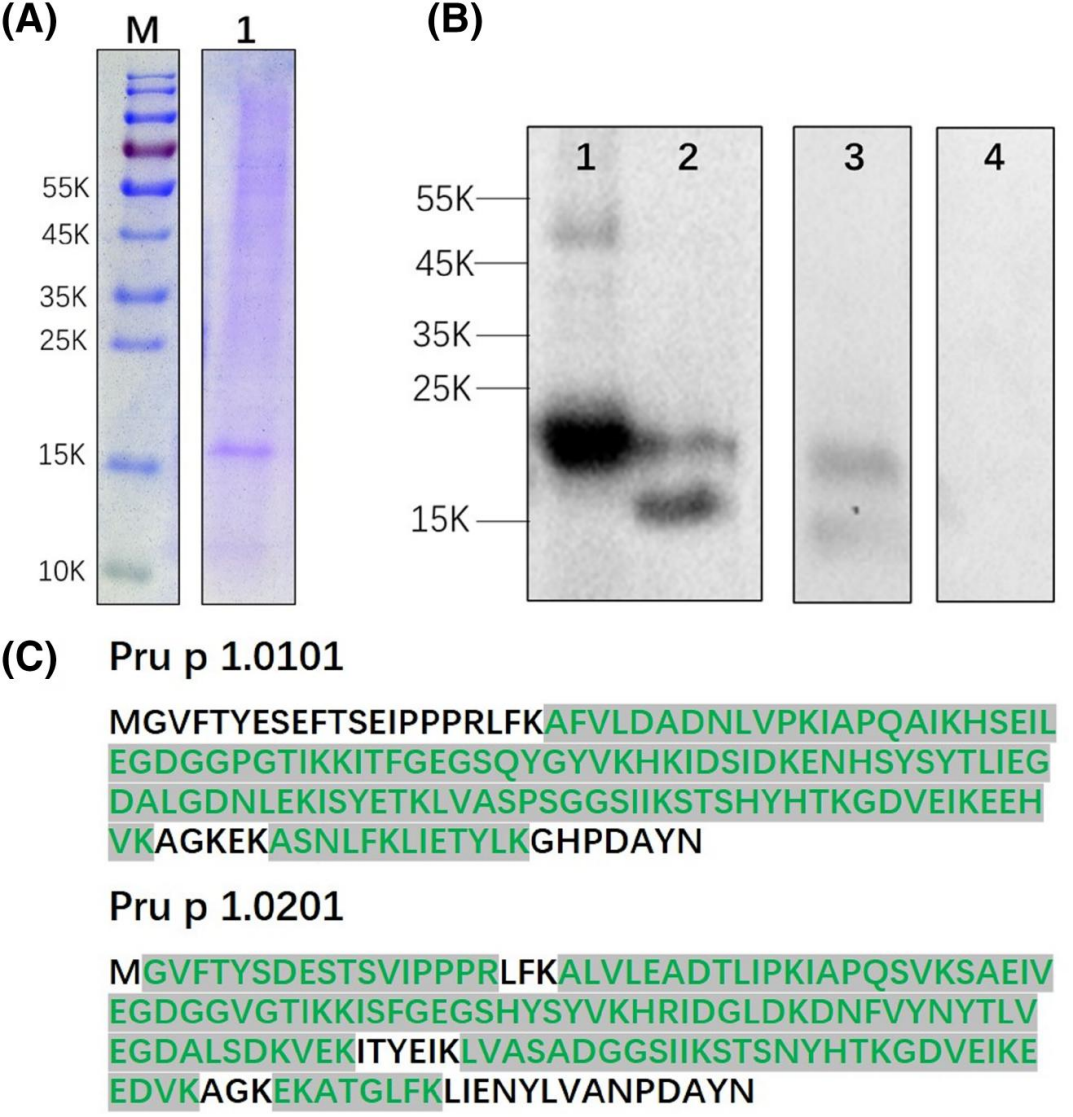
Identification of purified natural Pru p 1 by monoclonal antibody affinity. (A) Sodium dodecyl sulfate–polyacrylamide gel electrophoresis of the purified nPru p 1 (2 μg). (B) Western blot of the purified Pru p 1. (1) rPru p 1.0101 (0.2 μg); (2) nPru p 1 (2 μg, purity is 76%); (3) peach peel extract (cv. “Jin Shuo,” 6.5 μg); (4), BSA (0.2 μg). (C) Mature protein sequence, verified peptides by mass spectra fingerprint shown in green

The natural Pru p 1 purified through affinity chromatography was confirmed by immunoblot and LC‐MS (Figure 3). SDS‐PAGE and Western blot gave a band at 17 kDa (Figure 3A,B). Peptide spectrum matching gave the sequence coverage of 75.0% for Pru p 1.0101 and 83.1% for Pru p 1.0201 (Figure 3C).

### 3.4 Quantification of Pru p 1 in 83 peach varieties

**FIGURE 4:**
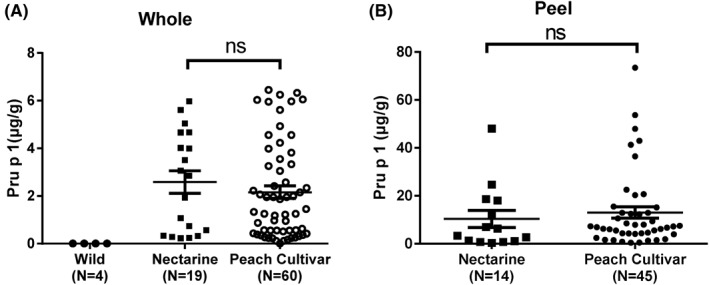
Comparison of Pru p 1 content in different varieties. (A) Pru p 1 content in whole fruits of wild peach, nectarines, and peach varieties. (B) Pru p 1 content in peel of nectarines and peach varieties. Difference between groups was assessed by Kruskal–Wallis nonparametric test followed by Dunn’s multiple comparison test (A); ns, not significant. Data expressed as mean ± SE

Even though Pru p 1 was generally low in the 83 cultivars, the variation ranged from 0.12 to 6.45 μg/g in whole fruit. There was an undetectable level of Pru p 1 content in four individual plants of the wild peach variety used for rootstock, and it was higher in nectarine (2.58 μg/g) than in pubescent peaches (2.16 μg/g) (Figure 4A). Pru p 1 was mainly concentrated in the peel (0.20–73.44 μg/g), less in the pulp (0.05–9.62 μg/g). There was no significant difference in Pru p 1 content between peel from nectarine and peach cultivars (shown in Figure 4B). Variety group, ripening date, and aroma intensity all had no significant effect on Pru p 1 (*p* > 0.05). Representative hypoallergenic Pru p 1 varieties were “Zi Xue Tao,” “Wu Yue Xian,” “May Fire,” and the high Pru p 1 allergen varieties were “Zhong You 7,” “Zao Zhen Bao,” “Chun Lei.”

### 3.5 Immunoblot of 10 representative varieties

**FIGURE 5:**
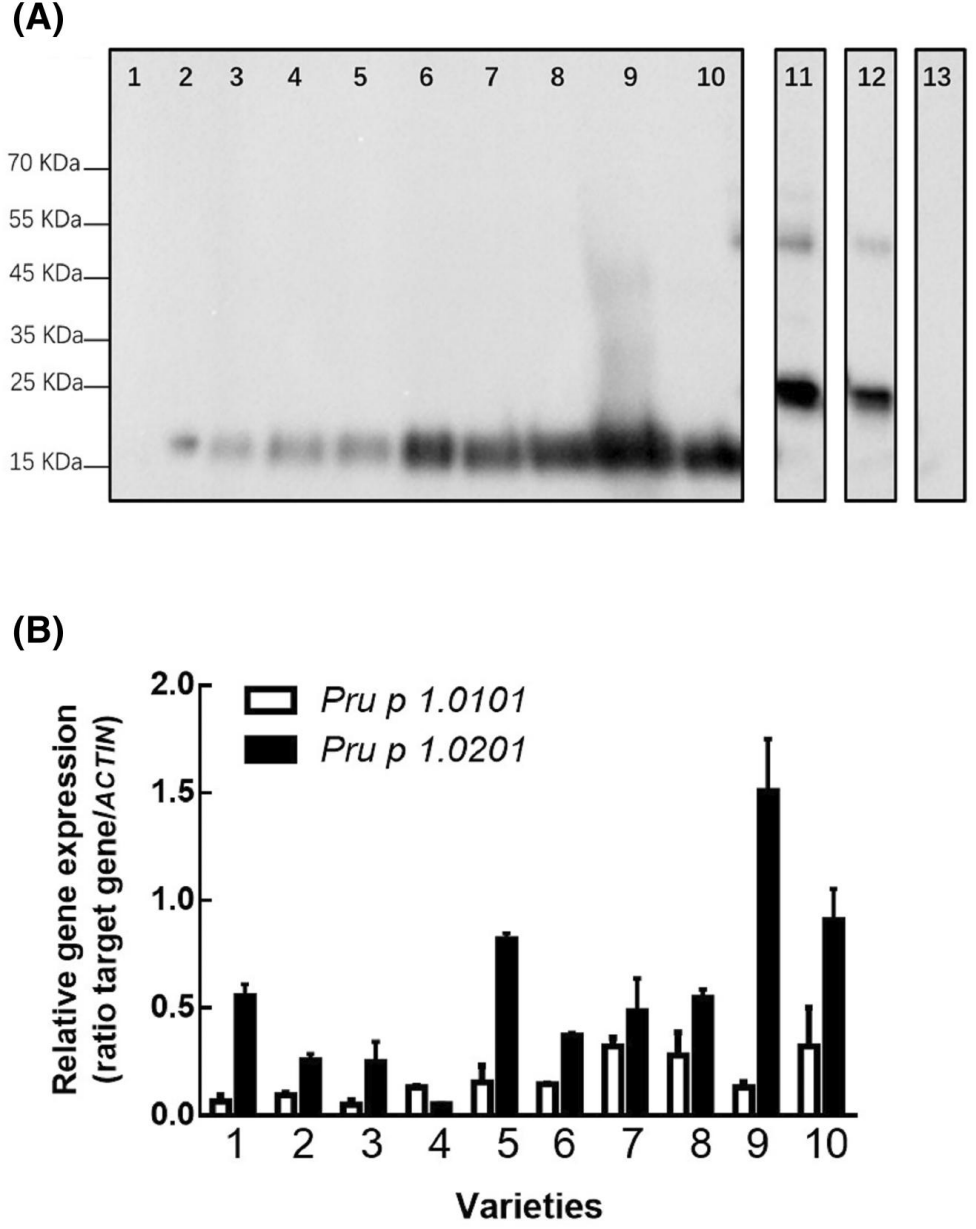
Immunoblot and quantitative polymerase chain reaction (qPCR) of 10 representative low/medium/high Pru p 1varieties. (A) (1) “Mao Tao 1”; 2–4: low Pru p 1 group. (2) “Zi Xue Tao”; (3) “Wu Yue Xian”; (4) “Nan Shan Tian Tao”; 5–7: medium Pru p 1 group. (5) “Xue Bu Dai”; 6), “Chi Yue”; (7) “Yuan Meng”; 8–10: high Pru p 1 group. (8) “Zao Zhen Bao”; (9) “Chun Lei”; (10), “Zhong You 7”; (11) rPru p 1.01 (0.25 μg); (12) rPru p 1.02 (0.1 μg); (13), BSA (0.1 μg). (B) qPCR results of *Pru p 1.0101* and *Pru p 1.0201* in 10 selected varieties

Ten varieties representing wild, low/medium/high‐Pru p 1 content groups based on the sELISA results were immunoblotted. Figure 5A shows that Pru p 1 was not detected in wild varieties. In peach and nectarine varieties, the 17 kDa bands were stronger with increasing Pru p 1 content, which indicated that immunoblot results were generally consistent with sELISA. Specific details, including concentration of peach crude extract, quantitative concentration of Pru p 1 and quantities of samples added are given in Table S2.

### 3.6 qPCR of *Pru p 1.0101* and *Pru p 1.0201* in 10 selected varieties

*Pru p 1.0101* and *Pru p 1.0201* expression levels were basically consistent with their Pru p 1 protein quantification: the increase in gene expression was in line with increase in content. Three representative varieties with low Pru p 1 content, “Zi Xue Tao,” “Wu Yue Xian,” and “Nan Shan Tian Tao” also had low *Pru p 1.0101* and *Pru p 1.0201* expression, while *Pru p 1* gene expression was high in varieties with relatively high Pru p 1 content, about four times higher than in low content varieties. In addition, the expression of *Pru p 1.0201* was higher than *Pru p 1.0101* in 10 tested varieties, except “Nan Shan Tian Tao” (Figure 5B).

## 4 DISCUSSION

sELISA is commonly used in fruit allergen quantification, for orange, apple, and peach.^15^, ^17^, ^18^, ^19^, ^25^ This method is advantageous in that it reacts directly with allergens in food and is highly sensitive and accurate. Owing to the similar epitopes of Pru p 1.01 and Pru p 1.02, our method was developed using a monoclonal (A0‐A7‐G11) and two polyclonal antibodies (P1 and P2), able to effectively identify these two major isoforms to determine total Pru p 1 level in peach fruit. Results from ELISA showed that the binding capacity of mAb A0‐A7‐G11 to the target antigens was high, but very low to other antigens (Table 2). The recovery rate, CVs, intra‐ and inter‐assay precision were acceptable (Table 3), with the quantification range determined as 4–32 ng/ml (Figure 2). These results demonstrate the development of ELISA with satisfactory sensitivity, accuracy, precision, reproducibility, and specificity for the detection of Pru p 1 in fresh peach. Because Pru p 1 is unstable, we took measures to minimize the degradation: the fruit was ground to powder in liquid nitrogen, and the dry powder stored at −40°C; protein extraction was performed in a 4°C refrigerator, and EDTA and diethyldithiocarbamate in the extraction buffer prevents degradation from allergens/proteins; samples were tested within 2 days after extraction.

Quantification of Pru p 1 in the peach and nectarine of 83 varieties collection ranged from 0.12 to 6.45 μg/g of FW: a previous report gave values between 0.01 and 0.18 μg/g of FW in whole fruit, 0.14 and 1.76 μg/g of FW in the peel for a limited number of varieties.^14^ PR‐10 proteins in other fruits had also been quantified, for example, Mal d 1 levels in apple ranging from 0.5 to 15 μg/g of FW, 3.8 to 72.5 μg/g of FW in the pulp or 0.71 to 20.17 μg/g FW in fresh fruits, and 5.95 to 200 μg/g FW in stored fruits.^15^, ^26^ It seems that the content of Mal d 1 in apples is much higher than Pru p 1 in peaches, which may explain the higher incidence of Mal d 1 allergy.

In China, there are three common sensitization patterns to peach allergen component: monosensitization to Pru p 1, cosensitization to Pru p 3 and Pru p 4, and monosensitization to Pru p 3.^7^, ^18^ Most peach allergic patients in China are positive to Pru p 3 due to primary sensitization to Pru p 3 and cross‐reactive to Artemisia pollen LTPs.^3^, ^7^, ^27^ The frequency of reactivity to Pru p 1 and Pru p 1 sIgE level is much lower than that of Pru p 3 in China.^3^, ^7^ This phenomenon may be related to the following two factors: first, a low chance of sensitization to birch pollen allergen PR‐10 that cross‐reacts with Pru p 1,^28^ due to the very limited number of birch trees in densely populated cities in Northern China and the virtual absence of birch trees in Southern China; second, the Pru p 1 content in peach fruit is very low.

In previous research we found that Pru p 3 is generally low in ancient and early‐maturing red flesh peach varieties.^19^ From an evolutionary point of view, Pru p 3 content was very low in the “Mao Tao” primary wild peaches and some local landraces (old varieties cultivated in a small area) of red‐fleshed peaches, and higher in most modern peach varieties, especially late‐ripening yellow‐fleshed varieties. A similar phenomenon is also found in the quantification of Pru p 1. Wild peaches and ancient red‐fleshed peaches such as “Zi Xue Tao,” and nectarines such as “May Fire,” usually contained very low Pru p 1. In contrast, some high quality fruit varieties with strong aroma and high sugar content such as “Jin Hua,” “Jin Shuo,” contained high concentration of Pru p 1 and Pru p 3. Therefore, artificial breeding programs seem to be developing in the direction of producing a high potential of allergen sensitization.

The expression of allergen encoding genes is affected by many factors. We have previously found that Pru p 3 content is related to ripening date, sugar content, and aroma, while this correlation was not identified in Pru p 1.^19^ Our quantification data showed that most nectarines contained high levels of Pru p 1, some up to 5.97 μg/g. Softening in these nectarines was slow, possibly due to the hydrolysis effect of Pru p 1.^29^

Unfortunately, the reduced levels of Pru p 1 do not always coincide with low Pru p 3. For example, nectarines such as “Zhong You 7,” “Hu You 277/278,” and “Nan Fang Jin Mi,” have been found to contain low Pru p 3 but high Pru p 1.^19^ This phenomenon is similar to Mal d 1 and Mal d 3 content in apple.^26^ Considering fruit quality, it is noticeable that those considered to be of good quality (strong aroma, high sugar content, and nutritional value) are usually accompanied with high allergen levels, as in peach and apricot.^19^, ^30^ Polyphenol oxidase activity and polyphenol content may be involved in decreasing Mal d 1 expression.^31^ Plant genetic factors also play an important role in fruit allergenicity, owing to the variations in expression of allergen isoforms.^26^, ^32^ Environmental factors also affect allergenicity. For example, light and fruit load (expressed as fruit number per square centimeter of trunk sectional area) affect the gene expression of *Pru p 1.0101* or *Pru p 1.0201* at the transcription level, which may ultimately affect the expression of Pru p 1.^32^

Pru p 1 is a pathogenesis‐related protein (PR‐protein), which means that pathogen attack, such as wounding, microbial infection, fungal infection, or environmental stress like light radiation, could induce or up‐regulate PR‐protein synthesis, especially on the outside of the organ, such as fruit peel.^2^, ^33^, ^34^ Significant reduction of the expression of *Pru p 1* genes has been observed in the peel rather in the pulp by fruit bagging with opaque paper.^35^

The level of *Pru p 1* in peel has been found to be more than 50 times that in the pulp.^24^ A previous study has shown that *Pru p 1.01* and *Pru p 1.02* gene expression are predominant and constitutively expressed with a maximum peak of expression in the S2 phase, and vary greatly in different cultivars in mature fruits.^32^ Here, we also found variation in its expression, with *Pru p 1.02* predominant, especially in the ancient varieties “Mao Tao” and “Xue Bu Dai.” In apple, a similar phenomenon has been found, with differing expression of three major Mal d 1 isoforms, Mal d 1.02 being the most highly expressed isoform.^26^ Gene expression by qPCR is only indicative of possible higher protein presence: our initial aim to quantify Pru p 1 .01 and Pru p 1.02 and correlated with the gene expression was not successful. Till now, no protein quantification to the isoallergen level by Sandwich ELISA in fruit has been available, perhaps requiring mass spectrometry aided with sensitive isoform‐specific peptide markers.

Our aim was to screen for hypoallergenic peach varieties, to allow breeders and growers to produce fruit with lower allergenic potential, which might be tolerated by patients with peach allergy. Results from this study may have implications for medical diagnostics, immunotherapy, clinical research, and breeding schemes for new hypoallergenic cultivars. We also identified peach varieties with high levels of Pru p 1 and Pru p 3 allergens, such as “Jin Shuo,” “Jin Feng,” and “Zao Zhen Bao” (shown in Table S1), which could be used as source material for diagnosis and for purification of natural Pru p 1 and Pru p 3 allergens.

## 5 CONCLUSION

A large variation of Pru p 1 content, ranging from 0.12 to 6.45 μg/g in FW, was observed among 83 peach/nectarine varieties, and mainly concentrated in the peel. The content of Pru p 1 in the whole fruits of nectarines was slightly higher than that of peaches, and the content of the wild peach “Mao Tao” was the lowest. Although the reduced levels of Pru p 1 do not always coincide with low Pru p 3, the ancient and early ripening red flesh varieties usually contained low Pru p 1 and Pru p 3 like “Zi Xue Tao,” “Wu Yue Xian,” and “May Fire.” This knowledge will help breeders select hypoallergenic cultivars for agricultural production, as well as medical practitioners for clinical trials.

## CONFLICT OF INTEREST

The author declares that there are no conflict of interests.

## AUTHOR CONTRIBUTIONS

**Jing Jin:** Data curation (lead), formal analysis (lead), investigation (lead), methodology (lead), validation (lead), writing‐original draft (lead). **Kexin Gan:** Data curation (supporting), investigation (supporting). **Lan Zhao:** Investigation (supporting). **Huijuan Jia:** Conceptualization (supporting), funding acquisition (equal). **Yifan Zhu:** Investigation (supporting). **Xiongwei Li:** Conceptualization (supporting), funding acquisition (equal), writing‐review and editing (supporting). **Zhaowei Yang:** Data curation (supporting), investigation (supporting). **Zhengwen Ye:** Resources (supporting). **Ke Cao:** Resources (supporting). **Zhiqiang Wang:** Resources (supporting). **Mingliang Yu:** Resources (supporting). **Yuyan Zhang:** Resources (supporting). **Zhisheng Ma:** Resources (supporting). **Hangkong Liu:** Resources (supporting). **Pere Arus:** Conceptualization (supporting), resources (supporting), writing‐review and editing (supporting). **Jaap Akkerdaas:** Conceptualization (supporting), formal analysis (supporting), writing‐original draft (supporting). **Zhongshan Gao:** Conceptualization (lead), data curation (equal), formal analysis (equal), funding acquisition (equal), supervision (lead), writing‐original draft (equal). **Ronald van Ree:** Conceptualization (supporting), methodology (supporting), supervision (supporting), writing‐review and editing (supporting).

## Supporting information

Supplementary Material

## References

[1] Gil MI , Tomás‐Barberán FA , Hess‐Pierce B , Kader AA . Antioxidant capacities, phenolic compounds, carotenoids, and vitamin C contents of nectarine, peach, and plum cultivars from California. J Agric Food Chem. 2002;50(17):4976‐4982. 12166993 10.1021/jf020136b

[2] Matricardi PM , Kleine‐Tebbe J , Hoffmann HJ , et al. EAACI molecular allergology user's guide. Pediatr Allergy Immunol. 2016;27(suppl 23):1‐250. 27288833 10.1111/pai.12563

[3] Gao ZS , Yang ZW , Wu SD , et al. Peach allergy in China: a dominant role for mugwort pollen lipid transfer protein as a primary sensitizer. J Allergy Clin Immunol. 2013;131(1):222‐224. 22939759 10.1016/j.jaci.2012.07.015

[4] Blanca M , Puche MV , Garrido‐Arandia M , et al. Correction: Pru p 9, a new allergen eliciting respiratory symptoms in subjects sensitized to peach tree pollen. PLoS ONE. 2020;15(3):e0230010. 32191737 10.1371/journal.pone.0230010 PMC7082028

[5] Giangrieco I , Ricciardi T , Alessandri C , et al. ENEA, a peach and apricot IgE‐binding protein cross‐reacting with the latex major allergen Hev b 5. Mol Immunol. 2019;112:347‐357. 31254775 10.1016/j.molimm.2019.05.007

[6] Gaier S , Marsh J , Oberhuber C , Rigby NM , Shewry PR . Purification and structural stability of the peach allergens Pru p 1 and Pru p 3. Mol Nutr Food Res. 2010;52(suppl 2):S220‐S229. 10.1002/mnfr.200700274 18384093

[7] Ma SK , Yin J , Jiang NN . Component‐resolved diagnosis of peach allergy and its relationship with prevalent allergenic pollens in China. J Allergy Clin Immunol. 2013;132:764‐767. 23791512 10.1016/j.jaci.2013.04.017

[8] Gamboa PM , Cáceres O , Antepara I , et al. Original article: two different profiles of peach allergy in the north of Spain. Allergy. 2007;62(4):408‐414. 17362252 10.1111/j.1398-9995.2006.01284.x

[9] Mothes N , Horak F , Valenta R . Transition from a botanical to a molecular classification in tree pollen allergy: implications for diagnosis and therapy. Int Arch Allergy Immunol. 2004;135(4):357‐373. 15583457 10.1159/000082332

[10] Ma SK , Wang RQ , Nie L , Yin J . Pollen‐food allergy syndrome in China. Food Agric Immunol. 2018;29(1):281‐293.

[11] Geroldinger‐Simic M , Zelniker T , Aberer W , et al. Birch pollen‐related food allergy: clinical aspects and the role of allergen‐specific IgE and IgG4 antibodies. J Allergy Clin Immunol. 2011;127(3):616‐622. 21251701 10.1016/j.jaci.2010.10.027

[12] Chen L , Zhang S , Illa E , et al. Genomic characterization of putative allergen genes in peach/almond and their synteny with apple. BMC Genom. 2008;9(1):543. 10.1186/1471-2164-9-543 PMC2621206 19014629

[13] Gao ZS , Zhou X , Yang ZW , et al. IgE‐binding potencies of three peach Pru p 1 isoforms. Mol Nutr Food Res. 2016;60(11):2457‐2466. 27374664 10.1002/mnfr.201500798

[14] Ahrazem O , Jimeno L , López‐Torrejón G , et al. Assessing allergen levels in peach and nectarine cultivars. Ann Allergy Asthma Immunol. 2007;99(1):42‐47. 17650828 10.1016/S1081-1206(10)60619-9

[15] Matthes A , Schmitz‐Eiberger M . Apple (*Malus domestica* L. Borkh.) allergen Mal d 1: effect of cultivar, cultivation system, and storage conditions. J Agric Food Chem. 2009;57(22):10548‐10553. 19845340 10.1021/jf901938q

[16] Elisabeth K , Vanessa K , Roberto LS , Klaus O , Wilfried S . Effect of the strawberry genotype, cultivation and processing on the Fra a 1 allergen content. Nutrients. 2018;10(7):57. 10.3390/nu10070857 PMC6073608 30004458

[17] Sancho AI , van Ree R , van Leeuwen A , et al. Measurement of lipid transfer protein in 88 apple cultivars. Int Arch Allergy Immunol. 2007;146(1):19‐26. 18087158 10.1159/000112499

[18] Gao ZS , Ma YT , Zhou X , et al. Quantification of peach fruit allergen lipid transfer protein by a double monoclonal antibody‐based sandwich ELISA. Food Anal Methods. 2015;9(4):823‐830.

[19] Jin J , Gao L , Zhao L , et al. Selection of Pru p 3 hypoallergenic peach and nectarine varieties. Allergy. 2020;75(5):1256‐1260. 31710093 10.1111/all.14102

[20] Daniela K , Gerhard B , Michaela SE . Impact of storage conditions on the apple allergen Mal d 1. Erwerbs‐Obstbau. 2012;54:177‐183.

[21] Li XW , Meng XQ , Jia HJ , et al. Peach genetic resources: diversity, population structure and linkage disequilibrium. BMC Genet. 2013;14(1):84. 24041442 10.1186/1471-2156-14-84 PMC3848491

[22] Micheletti D , Dettori MT , Micali S , et al. Whole‐genome analysis of diversity and SNP‐major gene association in peach germplasm. PLoS ONE. 2015;10(9):e136803. 10.1371/journal.pone.0136803 PMC4564248 26352671

[23] Wang L , Zhu G . Descriptors and Data Standard for Peach (*Prunus persica* L.). Beijing, China: China Agriculture Pres; 2005.

[24] Yang ZW , Ma YT , Chen L , et al. Differential transcript abundance and genotypic variation of four putative allergen‐encoding gene families in melting peach. Tree Genet Genomes. 2011;7(5):903‐916.

[25] Kiyota K , Kawatsu K , Sakata J , et al. Development of monoclonal antibody‐based ELISA for the quantification of orange allergen Cit s 2 in fresh and processed oranges. Food Chem. 2017;232(10):43‐48. 28490094 10.1016/j.foodchem.2017.03.140

[26] Sancho AI , Foxall R , Browne T , et al. Effect of postharvest storage on the expression of the apple allergen Mal d 1. J Agric Food Chem. 2006;54(16):5917‐5923. 16881695 10.1021/jf060880m

[27] Zhao L , Fu WY , Gao BY , et al. Variation in IgE binding potencies of seven Artemisia species depending on content of major allergens. Clin Transl Allergy. 2020;10:50. 33292509 10.1186/s13601-020-00354-7 PMC7677751

[28] Andersen M‐BS , Hall S , Dragsted LO . Identification of European allergy patterns to the allergen families PR‐10, LTP, and profilin from Rosaceae fruits. Clin Rev Allergy Immunol. 2011;41(1):4‐19. 19851893 10.1007/s12016-009-8177-3

[29] Zubini P , Zambelli B , Musiani F , Ciurli S , Bertolini P , Baraldi E . The RNA hydrolysis and the cytokinin binding activities of PR‐10 proteins are differently performed by two isoforms of the Pru p 1 peach major allergen and are possibly functionally related. Plant Physiol. 2009;150:1235‐1247. 19474212 10.1104/pp.109.139543 PMC2705045

[30] Hallmann E , Rozpara E , Słowianek M , Leszczyńska J . The effect of organic and conventional farm management on the allergenic potency and bioactive compounds status of apricots (*Prunus armeniaca* L.). Food Chem. 2019;279:171‐178. 30611476 10.1016/j.foodchem.2018.12.018

[31] Schmitz‐Eiberger M , Matthes A . Effect of harvest maturity, duration of storage and shelf life of apples on the allergen Mal d 1, polyphenoloxidase activity and polyphenol content. Food Chem. 2011;127(4):1459‐1464.

[32] Botton A , Andreotti C , Costa G , Ramina A . Peach (*Prunus persica* L. Batsch) allergen‐encoding genes are developmentally regulated and affected by fruit load and light radiation. J Agric Food Chem. 2009;57(2):724‐734. 19090764 10.1021/jf802709k

[33] Hoffmann‐Sommergruber K . Plant allergens and pathogenesis‐related proteins. Int Arch Allergy Immunol. 2000;122(3):155‐166. 10899758 10.1159/000024392

[34] Liu JJ , Ekramoddoullah AKM , Piggott N , Zamani A . Molecular cloning of a pathogen/wound‐inducible PR10 promoter from *Pinus monticola* and characterization in transgenic Arabidopsis plants. Planta. 2005;221(2):159‐169. 15609047 10.1007/s00425-004-1428-x

[35] Ma YT , Zhao XJ , Ren HW , et al. Significant reduction of the expression of peach (*Prunus persica* L. Batsch) allergen‐encoding genes by fruit bagging with opaque paper. J Agric Food Chem. 2018;66:4051‐4061. 29634265 10.1021/acs.jafc.8b00207

