# Supplementary material for: Peach allergen Pru p 1 content is generally low in fruit but with large variation in different varieties
Source: Clin Transl Allergy. 2021 May 14;11(3):e12034. doi: 10.1002/clt2.12034 (PMC8120414; doi:10.1002/clt2.12034)
Supplement: Supplementary file 1 — Supplementary Material [file CLT2-11-e12034-s001.docx]

**Supplementary Table 1. List of 83 core peach accessions, their basic fruit traits, Pru p 1 and Pru p 3 content.**

| **Code** | **Variety** | **Pedigree** | **Origin country** | **Fruit trait*** | **TSS (%)** | **Aroma** | | **Maturity time** | **Pru p 1 (ug/g FW±SD)** | **Pru p 3 (ug/g FW±SD)#** |
| --- | --- | --- | --- | --- | --- | --- | --- | --- | --- | --- |
| 1 | Mao Tao 1 | Wild peach for rootstock | Zhejiang province, China | PWR | 9.4 | L | | Early August | ND | 3.47±0.52 |
| 2 | Mao Tao 2 | Wild peach for rootstock | Zhejiang province, China | PYR | 11.9 | L | | Early August | ND | 8.53±0.44 |
| 3 | Mao Tao 3 | Wild peach for rootstock | Zhejiang province, China | PWR | 12.4 | L | | Middle August | ND | 11.35±0.59 |
| 4 | Mao Tao 4 | Wild peach for rootstock | Zhejiang province, China | PWR | 7.3 | L | | Middle August | ND | 14.35±0.12 |
| 5 | Da Hong Pao | Seedling | Wuhan, China | PRR | 14.0 | M | | Late June | ND | 7.82±0.56 |
| 6 | Zao Feng Huang | unknown | Zhejiang province, China | PWR | 12.5 | L | | Middle June | 0.12±0.03 | 26.25±1.48 |
| 7 | Xi Pu |  | Zhejiang province, China | PWR | 14.2 | H | | Late July | 0.12±0.04 | 26.71±2.53 |
| 8 | Qiu Yu Lu |  | Zhejiang province, China | PWR | 17.8 | H | | Middle August | 0.16±0.03 | 17.89±0.01 |
| 9 | May Fire | Seeding Selection of Armking | USA | NYR | 11.4 | L | | Late May | 0.22±0.06 | 3.54±0.17 |
| 10 | Hu Jing Mi Lu | Seedling of Hakuho | Jiangsu province, China | PWR | 14.7 | M | | Middle July | 0.22±0.05 | 25.43±1.22 |
| 11 | Zi Xue Tao | Landrace | Zhejiang province, China | PRR | 9.1 | L | | Late June | 0.23±0.06 | 7.12±0.13 |
| 12 | Okubo | Seedling of Hakuho | Japan | PWR | 13.14 | M | | Early July | 0.23±0.04 | 25.17±1.22 |
| 13 | Zhong You Tao 13 | unknown | Henan province, China | NYR | 12.1 | M | | Early June | 0.24±0.02 | 27.95±0.94 |
| 14 | Zhong Hua Shou Tao | Selection and breeding from the bud mutation of winter peach in north China | Shandong,China | PWR | 12.2 | H | | Early September | 0.25±0.07 | 47.76±1.91 |
| 15 | Qing Tao | Seedling | Zhejiang province, China | PWR | 17.7 | H | | Early September | 0.26±0.08 | 32.34±1.24 |
| 16 | Jin Xia You Pan | Xia Guang×‘NF’ | Jiangsu,China | NYF | 13.4 | L | | Early July | 0.28±0.07 | 10.89±0.72 |
| 17 | Wu Yue Xian | Landrace | Shanxi province, China | PRR | 9.0 | L | | Middle June | 0.31±0.05 | 8.02±0.28 |
| 18 | Zhong You Tao 11 | Zhong You Tao 5×SD9238(Rui Guang 3x May fire) | Henan province, China | NYR | 11.2 | M | | Late June | 0.32±0.07 | 7.18±0.42 |
| 19 | Armking | Palomar × Springtime | USA | NYR | 11.0 | L | | Early June | 0.33±0.07 | 6.68±1.50 |
| 20 | Qing Shui Bai Tao | unknown | Japan | PWR | 15.0 | H | | Middle July | 0.34±0.04 | 18.02±0.04 |
| 21 | Jing Yu | Okubo×Xing Jin You Tao | Beijing, China | PWR | 14.4 | L | | Middle July | 0.36±0.05 | 55.09±0.11 |
| 22 | Mei Shuo | ‘Jinyu' selfing | Hebei province, China | PWR | 12.3 | M | | Early June | 0.38±0.03 | 29.17±0.94 |
| 23 | Nan Shan Tian Tao | Landrace | Guangdong province, China | PWR | 18.0 | L | | Middle July | 0.42±0.07 | 34.78±1.31 |
| 24 | X1-4 | Yu Lu×Hu Jing Mi Lu | Zhejiang province, China | PWR | 13.0 | M | | Early July | 0.43±0.08 | 24.91±0.26 |
| 25 | Mei Shuai | Okubo×90-1(Ba Yue Cui×Jing Yu) | Hebei province, China | PWR | 12.6 | M | | Middle July | 0.51±0,.06 | 27.67±0.94 |
| 26 | Jin Xiu Huang Tao | Bai Hua ×Yun Shu 1 | Shanghai, China | PYR | 15.1 | M | | Early August | 0.55±0.05 | 21.14±0.52 |
| 27 | Jin Xiang | Bei Nong 2×60-27-7 | Shanghai, China | PYR | 15.6 | H | | Middle July | 0.55±0.07 | 18.58±0.50 |
| 28 | Chun Mi | 89-3-16（Zao Hong 2×Li He Pan Tao）×SD9238（Rui Guang 3×May Fire） | Henan province, China | PWR | 10.4 | M | | Early June | 0.55±0.06 | 21.91±0.16 |
| 29 | Li You 5 Hao | unknown | Zhejiang province, China | NYR | 9.3 | L | | Late June | 0.56±0.09 | 20.89±1.10 |
| 30 | Tai Gu Rou Tao | Landrace | Shanxi province, China | PWR | 17.4 | L | | Early August | 0.56±0.05 | 41.66±5.52 |
| 31 | Kawanakajima Hakuto | Found in White peach and Shang Hai Shui Mi mixed garden | Japan | PWR | 15.1 | M | | Early July | 0.56±0.06 | 24.94±0.64 |
| 32 | Huang Mei Gui |  | Zhejiang province, China | PYR | 11.0 | M | | Late July | 0.62±0.08 | 24.83±4.35 |
| 33 | Zhen Zhu Zao You Tao |  | Zhejiang province, China | NWR | 15.6 | L | | Late July | 0.74±0.08 | 23.38±2.33 |
| 34 | Mei Jin | ‘Jinyu' selfing | Hebei province, China | PYR | 14.6 | L | | Early July | 0.87±0.09 | 37.42±1.78 |
| 35 | Zheng Huang 3 Hao | Zao Shu Huang Gan×Feng Huang | Henan province, China | PYR | 6.2 | M | | Early July | 0.95±0.11 | 49.01±5.01 |
| 36 | Ling Can 1 Hao | Landrace | Zhejiang province, China | PWR | 17.9 | H | | Early September | 0.97±0.07 | 20.38±1.80 |
| 37 | Zhong You Tao 8 | Hong Shan Hu×Sunshine | Henan province, China | NYR | 14.5 | L | | Middle July | 1.07±0.09 | 7.85±0.30 |
| 38 | Mei Shuai | Okubo×90-1(Ba Yue Cui×Jing Yu) | Hebei province, China | PWR | 12.6 | M | | Middle July | 1.18±0.07 | 27.67±0.94 |
| 39 | Ling Shen 2 Hao | Landrace | Zhejiang province, China | PWR | 16.1 | L | | Early September | 1.22±0.14 | 21.11±1.07 |
| 40 | Xue Bu Dai | Landrace | Henan province, China | PRR | 12.0 | L | | Early July | 1.25±0.16 | 4.00±0.95 |
| 41 | Xue Xiang Lu | Bai Hua×Chu Xiang Mei | Jiangsu province, China | PWR | 11.3 | M | | Middle June | 1.25±0.12 | 22.09±0.96 |
| 42 | X1-7 | Yu Lu×Hu Jing Mi Lu | Zhejiang province, China | PWR | 10.8 | M | | Early July | 1.33±0.09 | 27.31±0.15 |
| 43 | Reddomun | Bai Feng×Bai Tao | Japan | PWR | 12.2 | M | | Early July | 1.45±0.20 | 24.48±0.67 |
| 44 | Meng Lu Shui Jing | Seedling | Zhejiang province, China | PWR | 15.5 | M | | Middle July | 1.86±0.06 | 21.88±0.16 |
| 45 | Rui Guang 2 | Jingyu x NJN76 | Beijing, China | NYR | 12.8 | M | | Late May | 1.93±0.09 | 16.77±1.40 |
| 46 | Xin Yu | Landrace | Zhejiang province, China | PWR | 14.9 | M | | Middle July | 1.95±0.29 | 26.77±6.17 |
| 47 | Zao Shang Hai Shui Mi | Seedling | Shanghai, China | PWR | 8.4 | M | | Middle June | 1.95±0.38 | 20.19±2.48 |
| 48 | Asama Hakuto | Bud mutation from Kouyou Hakuto | Japan | PWR | 16.2 | M | | Middle July | 1.95±0.22 | 28.28±0.23 |
| 49 | Pan Tao Wang | Early Red 2×Zao Lu Pan Tao | Henan province, China | PWF | 13.1 | M | | Early June | 1.99±0.14 | 8.13±0.13 |
| 50 | Zao Mei | Qing Feng×Zhao Xia | Beijing, China | PWR | 10.8 | M | | Late May | 2.06±0.32 | 14.33±0.36 |
| 51 | Xia Cui | Yu Hua 2 ×77- 1- 6((Bai Hua×Tachibana Wase)×Zhao Xia) | Jiangsu province, China | PWR | 12.4 | M | | Late June | 2.15±0.45 | 23.95±1.96 |
| 52 | Sunago Wase | Seedling | Japan | PWR | 10.6 | M | | Middle June | 2.22±0.81 | 31.50±0.55 |
| 53 | Qin Wang | Seedling from Okubo | Shaanxi province, China | PWR | 17.1 | L | | Middle July | 2.34±0.44 | 33.45±0.94 |
| 54 | Yan Hong | Seedling | Beijing, China | PWR | 15.9 | L | | Late July | 2.42±0.25 | 33.87±0.40 |
| 55 | Qiu Bai Tao | Seedling | Zhejiang province, China | PWR | 13.9 | L | | Late July | 2.57±0.13 | 35.01±1.28 |
| 56 | Zhong You Tao 4 | Ruiguang 16×May Fire | Henan province, China | NYR | 12.6 | M | | Early June | 2.85±0.35 | 13.62±0.65 |
| 57 | Yu Lu Pan Tao | Landrace | Shanghai, China | PWF | 15.0 | H | | Middle July | 3.05±0.33 | 49.92±7.07 |
| 58 | Zhong Nong Jin Hui | Rui Guang 2×Armking | Henan province, China | NYR | 12.8 | M | | Eraly June | 3.06±0.29 | 9.07±0.66 |
| 59 | Yuan Meng | Hu Jing Mi Lu×Hakuri | Zhejiang province, China | PWR | 13.1 | L | | Early August | 3.26±0.20 | 40.81±4.66 |
| 60 | Jin Yuan | Jin Xiu×75-1-3 | Shanghai, China | PWR | 17.7 | L | | Late July | 3.33±0.34 | 14.61±0.43 |
| 61 | Chao Li Chun | Ruiguang 3 x Mayfire | Beijing, China | NYR | 9.3 | L | | Late May | 3.50±0.09 | 13.53±0.09 |
| 62 | Sha Hong Tao | Bud mutation from Kurakato Wase | Shaanxi province, China | PWR | 15.6 | M | | Early July | 3.55±0.41 | 19.67±0.26 |
| 63 | Hong Sha Zi | seedling | Shaanxi, China | PWR | 11.8 | M | | Early June | 3.82±0.26 | 30.58±0.37 |
| 64 | Mei Gui Lu | Sunago Wase×Yu Hua Lu | Zhejiang province, China | PWR | 11.9 | M | | Early June | 3.98±0.81 | 44.86±2.12 |
| 65 | Nan Fang Jin Mi | (Sunred×Maravilha）1-15 x Shu Guang | Henan province, China | NYR | 14.9 | M | | Late May | 3.99±0.24 | 18.59±0.20 |
| 66 | Zi Jin Hong 1 Hao | Natural seed cultivated by embryo rescue | Jiangsu, China | NYR | 9.7 | L | | Early June | 4.02±0.47 | 20.12±0.57 |
| 67 | Da Guan 1 Hao | Selected from Nunomewase | Henan province, China | PWR | 9.3 | M | | Early June | 4.23±0.29 | 24.81±1.20 |
| 68 | Akatsuki | Bai Tao×Bai Feng | Japan | PWR | 12.8 | M | | Early July | 4.56±0.17 | 27.26±1.34 |
| 69 | Xin Hong | Landrace | Zhejiang province, China | PWR | 14.1 | M | | Early July | 4.56±0.44 | 25.20±2.99 |
| 70 | Ying Guang You Tao | unknown | Zhejiang province, China | NWR | 12.6 | M | | Late May | 4.66±0.32 | 23.42±0.91 |
| 71 | Zao Hong Lu | Armking×81-3-3 | Bejing,China | NWR | 10.5 | M | | Early June | 4.67±0.26 | 15.50±0.25 |
| 72 | Tai Yuan Shui Mi | Landrace | Shanxi province, China | PWR | 17.4 | L | | Early August | 4.93±0.29 | 41.66±5.52 |
| 73 | Hu You 277 | Rui Guang 3×May Fire | Shanghai, China | NYR | 9.9 | L | | Middle June | 5.04±0.25 | 5.42±0.15 |
| 74 | Hu You 278 | Rui Guang 3×May Fire | Shanghai, China | NYR | 10.7 | L | | Middle June | 5.61±0.66 | 4.02±0.84 |
| 75 | Yan Feng | Landrace | Zhejiang province, China | PWR | 14.3 | M | | Middle June | 5.61±0.47 | 36.60±1.74 |
| 76 | Kurakato Wase | -- | Japan | PWR | 10.0 | H | | Middle June | 5.96±0.62 | 41.33±0.21 |
| 77 | Zhong You Tao 7 |  | Henan province, China | NWR | 11.6 | H | Middle July | | 5.97±0.31 | 11.92±0.46 |
| 78 | Nunome Wase | Seedling | Japan | PWR | 11.2 | H | Early June | | 5.97±0.28 | 36.43±1.33 |
| 79 | Jin Shuo | Yingqing x Yangtao | Shanghai, China | PYR | 17.5 | H | Early September | | 6.03±0.85 | 57.89±3.50 |
| 80 | Zao Zhen Bao | unknown | Zhejiang province, China | PWR | 10.3 | M | Late June | | 6.05±0.44 | 39.84±0.37 |
| 81 | Jin Feng |  | Shanghai, China | PYR | 12,8 | M | Late July | | 6.25±0.39 | 55.41±0.45 |
| 82 | Da Zhen Bao |  | Zhejiang Province,China | PWR | 11.5 | M | Late June | | 6.33±0.58 | 43,22±0.37 |
| 83 | Chun Lei | Sunago Wase×Bai Xiang Lu | Henan province, China | PWR | 9.3 | M | Late May | | 6.45±0.63 | 18.43±1.30 |

*****The first letter refers to P-peach, N-Nectarine; the second letter refers to flesh color: W-white, Y-Yellow flesh, R-Red flesh; the third letter refers to fruit shape: F-Flat, R-round shape

‡ND= analyzed but no Pru p 1 content detected

# data from previous publication (Jin et al, Allergy, 2020) online supplementary Table

**Supplementary Table 2. Detailed information of 10 representative low/medium/high Pru p 1 varieties in figure 5.**

| **Pru p 1 level group** | **Serial number** | **Variety** | **Peach crude extract concentration (μg/ml)** | **Quantitative concentration of Pru p 1 (μg/ml)** | **Samples added quantities in lanes(μg)** |
| --- | --- | --- | --- | --- | --- |
| Wild peach | 1 | Mao Tao 1 | 31.92 | ND | 0.6 |
| Low | 2 | Zi Xue Tao | 87.86 | 0.23 | 1.8 |
|  | 3 | Wu Yue Xian | 130.86 | 0.31 | 2.6 |
|  | 4 | Nan Shan Tian Tao | 84.82 | 0.42 | 1.7 |
| Medium | 5 | Xue Bu Dai | 117.93 | 1.25 | 1.6 |
|  | 6 | Chi Yue | 78.73 | 1.45 | 2.8 |
|  | 7 | Yuan Meng | 141.90 | 3.26 | 4.0 |
| High | 8 | Zao Zhen Bao | 199.74 | 5.04 | 4.7 |
|  | 9 | Chun Lei | 333.69 | 6.45 | 1.5 |
|  | 10 | Zhong You 7 | 76.45 | 5.97 | 2.4 |

‡ND= analyzed but no Pru p 1 content detected
